# Synthesis and Antimicrobial Activities of 5-Arylidene-thiazolidine-2,4-dione Derivatives

**DOI:** 10.1155/2014/316082

**Published:** 2014-05-07

**Authors:** Ivanildo Mangueira da Silva, João da Silva Filho, Priscila Brandão Gomes da Silva Santiago, Micalyne Soares do Egito, Carlos André de Souza, Frederico Leite Gouveia, Rafael Matos Ximenes, Kêsia Xisto da Fonseca Ribeiro de Sena, Antonio Rodolfo de Faria, Dalci José Brondani, Julianna Ferreira Cavalcanti de Albuquerque

**Affiliations:** ^1^Department of Pharmacy, Federal University of Pernambuco, Avenida Professor Moraes Rego 1235, Cidade Universitária, 50670-901 Recife, PE, Brazil; ^2^Department of Antibiotics, Federal University of Pernambuco, Avenida Professor Moraes Rego 1235, Cidade Universitária, 50670-901 Recife, PE, Brazil

## Abstract

Antibiotic resistance is considered one of the world's major public health concerns. The main cause of bacterial resistance is the improper and repeated use of antibiotics. To alleviate this problem, new chemical substances against microorganisms are being synthesized and tested. Thiazolidines are compounds having many pharmacological activities including antimicrobial activities. For this purpose some thiazolidine derivatives substituted at position 5 in the thiazolidine nucleus were synthesized and tested against several microorganisms. Using a disc diffusion method, antimicrobial activity was verified against Gram-positive, Gram-negative, and alcohol acid resistant bacteria and yeast. The minimum inhibition concentrations (MIC) and minimum bactericidal concentrations (MBC) were determined. All derivatives showed antimicrobial activity mainly against Gram-positive bacteria, with MIC values ranging from 2 to 16 µg/mL.

## 1. Introduction

Thiazolidine is a class of compounds which merit special attention because it belongs to a group of substances with activity in medicinal chemistry. This nucleus is associated with antibacterial, antifungal, antiviral, antituberculosis, anticancer, and antiparasite biological activities [1–4]. The use of new synthetic methods and structure-activity relationship studies has made possible a broad study of new drugs with different actions. The computational search for possible mechanisms of 4-thiazolidinones anticancer activity has been studied together with the medical chemistry which permits the optimization of existing drugs [5].

Currently, very important groups of heterocyclic compounds such as thiazolidine-2,4-dione, 2-imino-4-thiazolidinone, 4-thioxo-thiazolidine-2,4-dione, and 2-thioxo-1,3-thiazolidine-4-one (rhodanine) have attracted the attention of researchers in their search for new agents with specific pharmacological properties [6–14].

Multiple antibiotic resistant bacteria represent a challenge in the treatment of infections. It is imperative, therefore, that new substances with antimicrobial properties be found to fight these microorganisms [15]. To be considered a bacterium resistant to a certain antibiotic, the microorganism should be able to grow in vitro when subjected to an inhibitory concentration equal to that obtained in the blood. However, the concentration of several antibiotics in the bloodstream can be much lower than that achieved by the same antibiotic in other body tissues or fluids. Thus, a bacterium could be “resistant” to a certain antibiotic when it is present in the bloodstream but “sensitive” when it is in the urinary tract or vice versa [16].

Bacteria become resistant to chemotherapeutic agents by three main mechanisms: destruction or inactivation of the drug, prevention of the penetration of the target site within the microbe, and alteration of drug target sites. There may be variations in these mechanisms [17]. With increasing bacterial resistance to antibiotics, attention has become focused on the development of new derivatives to be used as antimicrobial therapy in infection control [18]. Antibiotics are substances produced synthetically by bacteria and fungi with the function of suppressing the growth of microorganisms [19]. Currently, new antibiotics are needed for the treatment of multidrug resistant bacteria. The clinical use of new drugs has decreased since the 1980s, due to a reduction in the discovery of new, more efficient, and less toxic drugs by pharmaceutical companies around the world. Other research groups are worried about the rise in recurrence of many infectious diseases and the lack of new drugs and development of new antimicrobial products in the face of increasing resistance to existing agents [20].

The literature reports on the results of a number of biological activities when the substituents and their positions on the thiazolidine ring are changed [21]. In this case, medicinal chemistry is an important aid in the discovery of new active molecules using small heterocyclic rings to increase the biological activity of certain nuclei [22]. Due to the importance of the core of the thiazolidine ring, eleven compounds have been synthesized by our research group, some products already known and others unknown by introducing arylidene groups at the position 5 of the thiazolidine ring in order to test the antimicrobial activity of each compound against nine different microorganisms.

## 2. Material and Methods

### 2.1. Chemistry

The chemical reagents were supplied by Sigma-Aldrich (USA) and were used without further purification. Purity of the compounds was checked using thin layer chromatography (TLC) plates (silica gel G) in the appropriated system for each compound. The spots were located with short (254 nm)/long (365 nm) UV wavelength.

**Scheme 1:**
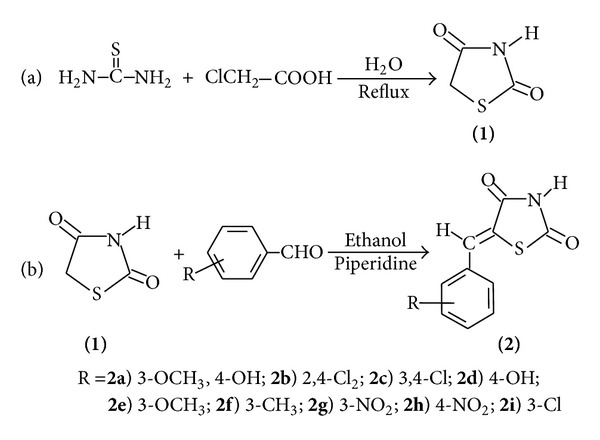
Substituent in the synthesis of 5-arylidene-thiazolidine-2,4-dione (**2a**–**i**).

All melting points were measured in a capillary tube on a Quimis apparatus. Infrared spectra of 1% KBr pellets were recorded using a Bruker IFS66 spectrometer. ^1^H NMR and ^13^C NMR spectra were measured on a VARIAN VNMRS 400-MR, using 400 MHz for ^1^H and 75.4 MHz for ^13^C in CDCl_3_ and acetone-d_6_ maintained at 25°C using Me_4_Si (TMS) as an internal standard. The chemical shifts were reported in *δ* units and the coupling constants (*J*) were reported in hertz. The following abbreviations were used to indicate the peak multiplicity: s (singlet), d (doublet), dd (double doublet), t (triplet), and m (multiplet). C, H, N, and S analyses were performed with a Carlo Erba elemental analyzer, model EA1108. Mass spectra were recorded on a Varian MAT 711 spectrometer at an electron impact of 70 eV. The synthesized compounds are shown in Scheme 1, showing the respective substituent.

### 2.2. Procedure for Preparation of the Synthesis of Thiazolidine-2,4-dione (**1**)

Compound (**1**) (thiazolidine-2,4-dione) was synthesized by refluxing monochloroacetic acid and thiourea in water. This compound was synthesized according to a published procedure [16]. Molecular formula C_3_H_3_O_2_NS; yield 78%; mp 118–120°C; Rf 0.48 (0.9 : 0.1 CHCl_3_/MeOH). Recrystallization: water.

### 2.3. General Method for the Synthesis of 5-Arylidene-thiazolidine-2,4-dione (**2a**–**i**)

The compound (**2a**–**i**) was synthesized from a mixture of thiazolidine-2,4-dione (**1**) (2.5 g, 21.36 mmol), aldehyde (21.36 mmol), piperidine (14.11 mmol), and ethanol (150 mL). The reaction mixture was heated under reflux and continuously stirred for a period of 8-9 h. The course of the reaction was monitored by TLC. The reaction mixture was poured into water and acidified with acetic acid. The resulting precipitate was filtered off and recrystallized from acetic acid to give (**2a**–**i**).

### 2.4. Microbiological Activity

Gram-positive, Gram-negative, and alcohol acid resistant bacteria and yeast were selected for the examination of antimicrobial activity in vitro. For the evaluation of the antimicrobial activity, first a disc diffusion test was used to screen the antimicrobial activity of all compounds. The compounds that had inhibition zones greater than 10 mm of diameter were submitted to a second test for the determination of minimum inhibitory concentration (MIC) and minimum bactericidal concentration (MBC). The tests were performed as follows.

#### 2.4.1. Disk Diffusion Method

Antimicrobial activity was evaluated by disc diffusion method according to Bauer et al. [23] against Gram-positive bacteria (*Staphylococcus aureus*—DAUFPE 01,* Micrococcus luteus*—DAUFPE 06,* Bacillus subtilis*—DAUFPE 16, and* Enterococcus faecalis*—DAUFPE 138), Gram-negative bacteria (*Pseudomonas aeruginosa*—DAUFPE 39,* Escherichia coli*—DAUFPE 224, and* Serratia marcescens*—DAUFPE 398), acid alcohol resistant bacteria (*Mycobacterium smegmatis*—DAUFPE 71), and yeast (*Candida albicans*—DAUFPE 1007) obtained from the culture collection of the Department of Antibiotics at the Federal University of Pernambuco (UFPE), Brazil.

Paper discs (Whatman number 2) with 6 mm diameters were impregnated with 20 *μ*L of a 15,000 *μ*g/mL solution of the synthesized compounds and dissolved in DMSO. The discs were then placed in medium sown with one of the microorganisms. The following standard drugs were used as controls: ketoconazole (Neoquímica, Brazil), for yeast and cefalexin (Eurofarma, Brazil) and kanamycin (Centro de Controle e Produtos para Diagnósticos—CECON, Brazil) for bacteria. The tests were carried out 3 times, with the results expressed (in millimeters) as mean ± SEM of the diameters of the inhibition zones formed around the discs. The negative control test was carried out with DMSO soaked discs.

#### 2.4.2. Minimum Inhibitory Concentration (MIC) and Minimum Bactericidal Concentration (MBC)

A multiwell plate (96 wells) was used to determine the minimum inhibitory concentration (MIC) following the criteria adopted by the Clinical and Laboratory Standards Institute (CLSI) [24]. Tests were carried out in Müeller-Hinton broth and Sabouraud dextrose broth (Difco, USA) at pH 7.4 and the twofold serial dilution technique was applied. A 1,280 *μ*g/mL stock solution was prepared from the product. A standardized suspension of microorganisms was prepared for use with a 0.5 tube on the McFarland scale. The plate was incubated for 18 hours and thereafter an indicator dye resazurin (Sigma-Aldrich, USA) was applied to show if there was microbial growth in the well. MIC was determined as the concentration of the last well where there was no microbial growth. From this experiment, the content of the wells was sown on plates with Müeller-Hinton agar medium to establish the minimum bactericidal concentration (MBC), which is the concentration where there is no colony growth. All analyses were performed in triplicate.

## 3. Result and Discussion

### 3.1. Synthesis

*5-(3-Methoxy-4-hydroxy-arylidene)-thiazolidine-2,4-dione (**2a**)*. Yield 70%; mp 260°C; Rf 0.50 (CHCl_3_/MeOH 9 : 1). Recrystallization: ethanol; IR (KBr 1%, *ν*_max⁡_ cm^−1^) 1566 (C=C); 1730–1670 (C=O). ^1^H NMR (CDCl_3, _300 MHz, *δ* ppm): 8.48 (s 1H, NH); 7.71 (s 1H, CH=); 3.77 (s 3H, OCH_3_); 6.02 (s 1H, OH). 6.92 (s 1H_(2)_); 6.86 (d 1H_(5)_ *J* = 8.33); 6.91 (d 1H_(6)_ *J* = 8.34); ^13^C NMR (Acetone-d_6_, 75.4 MHz, *δ* ppm): 169.81 (C=O_2_); 166.89 (C=O_4_); 119.55 (C_5_, heterocycle); 143.28 (CH=); 56.50 (OCH_3_); 129.20 (C_1_); 112.58 (C_2_), 148.06 (C_3_); 144.76 (C_4_); 115.56 (C_5_); 122.30 (C_6_). Anal. Calcd. for C_11_H_9_NO_4_S: C, (52.58%); H, (3.61%); N, (5.57%). Found: C, 53.01%; H, 3.83%; N, 5.25%. HRMS^+^: calcd 251.0252; found 251.0253.

*5-(2,4-Dichloro-arylidene)-thiazolidine-2,4-dione (**2b**)*. Yield 65%; mp 203°C; Rf 0.51 (CHCl_3_/MeOH 9.6 : 0.4). Recrystallization: ethanol; IR (KBr 1%, *ν*_max⁡_ cm^−1^) 1570 (C=C); 1737–1675 (C=O). ^1^H NMR (CDCl_3, _300 MHz, *δ* ppm): 8.50 (s 1H, NH); 7.35 (s 1H, CH=); 7.55 (s 1H, H_(3) _7.44 (d 1H, H_(5)_ *J* = 7.90); 7.39 (d 1H (H_(6)_ *J* = 7.90). ^13^C NMR (Acetone-d_6_, 75.4 MHz, *δ* ppm): 169.85 (C=O_2_); 166.86 (C=O_4_); 120.20 (C_5_ heterocycle); 140.23 (CH=); 133.61 (C_1_); 134.84 (C_2_); 129.52 (C_3_); 134.93 (C_4_); 127.52 (C_5_); 135.80 (C_6_). Anal. Calcd. for C_10_H_5_Cl_2_NO_2_S: C, 43.81%; H, 1.84%; N, 5.11%. Found: C, 44.01%; H, 1.96%; N, 5.05%. HRMS^+^: calcd 272.9418; found 272.9419.

*5-(3,4-Dichloro-arylidene)-thiazolidine-2,4-dione (**2c**)*. Yield 59%; mp 174°C; Rf 0.50 (CHCl_3_/MeOH 9.5 : 0.5). Recrystallization: ethanol; IR (KBr 1%, *ν*_max⁡_ cm^−1^) 1571 (C=C); 1735–1676 (C=O). ^1^H NMR (CDCl_3, _300 MHz, *δ* ppm): 8.50 (s 1H, NH); 7.77 (s 1H, CH=); 7.52 (s 1H, H_(2)_; 7.38 (d 1H, H_(5)_ *J* = 8.91); 7.52 (d 1H (H_(6)_ *J* = 8.92). ^13^C NMR (Acetone-d_6_, 75.4 MHz, *δ* ppm): 169.86 (C=O_2_); 166.89 (C=O_4_); 119.17 (C_5_ heterocycle); 143.13 (CH=); 134.42 (C_1_); 129.49 (C_2_); 133.15 (C_3_); 131.79 (C_4_); 130.71 (C_5_); 127.26 (C_6_). Anal. Calcd. for C_10_H_5_Cl_2_NO_2_S: C, 43.81%; H, 1.84%; N, 5.11%. Found: C, 43.91%; H, 1.97%; N, 5.07%. HRMS^+^: calcd 272.9418; found 272.9419. 

*5-(4-Hidroxy-arylidene)-thiazolidine-2,4-dione (**2d**)*. Yield 45%; mp 149-150°C; Rf 0.49 (CH_2_Cl_2_/Hex. 5 : 5). Recrystallization: methanol; IR (KBr 1%, *ν*_max⁡_ cm^−1^) 1570 (C=C); 1734–1677 (C=O). ^1^H NMR (CDCl_3, _300 MHz, *δ* ppm): 8.49 (s 1H, NH); 7.54 (s 1H, CH=); 6.40 (s 1H, OH); 7.31 (d 1H, H_(2)_–H_(6)_ *J* = 8.65); 7.21 (d 1H, H_(3)_–H_(5)_ *J* = 8.65). ^13^C NMR (Acetone-d_6_, 75.4 MHz, *δ* ppm): 169.86 (C=O_2_); 166.89 (C=O_4_); 119.55 (C_5_ heterocycle); 140.25 (CH=); 129.15 (C_1_); 129.15 (C_2_)–(C_6_); 115.96 (C_3_)–(C_5_); 157.71 (C_4_). Anal. Calcd. for C_10_H_7_NO_3_S: C, 54.29%; H, 3.19%; N, 6.33%. Found: C, 54.93%; H, 3.23%; N, 6.11%. HRMS^+^: calcd 221.0147; found 272.0148. 

*5-(4-Methoxy-arylidene)-thiazolidine-2,4-dione (**2e**)*. Yield 40%; mp 210–212°C; Rf 0.48 (CHCl_3_/MeOH 9.6 : 0.4). Recrystallization: CHCl_3_; IR (KBr 1%, *ν*_max⁡_ cm^−1^) 1575 (C=C); 1734–1676 (C=O). ^1^H NMR (CDCl_3, _300 MHz, *δ* ppm): 8.49 (s 1H, NH); 7.74 (s 1H, CH=); 3.65 (s 3H, OCH_3_); 7.35 (d 1H, H_(2)_–H_(6)_ *J* = 8.65); 6.75 (d 1H (H_(3)_–H_(5)_ *J* = 8.65). ^13^C NMR (Acetone-d_6_, 75.4 MHz, *δ* ppm): 169.86 (C=O_2_); 166.89 (C=O_4_); 119.50 (C_5_ heterocycle); 142.55 (CH=); 55.67 (OCH_3_); 128.42 (C_1_); 129.37 (C_2_)–(C_6_); 114.32 (C_3_)–(C_5_); 160.31 (C_4_). Anal. Calcd. for C_11_H_9_NO_3_S: C, 56.16%; H, 3.86%; N; 5.95%. Found: C, 56.98%; H, 3.99%; N, 6.45%. HRMS^+^: calcd 235.0303; found 235.0303. 

*5-(3-Methyl-arylidene)-thiazolidine-2,4-dione (**2f**)*. Yield 52%; mp 195-196°C; Rf 0.50 (Hex./Ethyl acetate 8.5 : 1.5). Recrystallization: acetic acid; IR (KBr 1%, *ν*_max⁡_ cm^−1^) 1573 (C=C); 1730–1739 (C=O); ^1^H NMR (CDCl_3, _300 MHz, *δ* ppm): 8.49 (s 1H, NH); 7.73 (s 1H, CH=); 2.36 (s, 3H CH_3_); 7.25 (s 1H H_(2)_); 6.95 (d 1H H_(4)_ *J* = 7.71); 7.31 (t, 2H H_5_,  *J* = 7.72); 7.31 (d 1H H_6_ *J* = 7.52). ^13^C NMR (Acetone-d_6_, 75.4 MHz, *δ* ppm): 169.86 (C=O_2_); 167.89 (C=O_4_); 119.55 (C_5_ heterocycle); 143.18 (CH=); 21.20 CH_3_; 136.18 (C_1_); 128.93 (C_2_); 133.87 (C_3_); 126.60 (C_4_); 129.51 (C_5_); 125.93 (C_6_). Anal. Calcd. for C_11_H_9_NO_2_S: C, 60.26%; H, 4.14%; N, 6.39%. Found: C, 60.66%; H, 4.45%; N, 6.11%; HRMS^+^: calcd 219.0354; found 272.0353. 

*5-(3-Nitro-arylidene)-thiazolidine-2,4-dione (**2g**)*. Yield 80%; mp 250°C; Rf 0.51 (CHCl_3_/MeOH 9.6 : 0.4). Recrystallization: acetic acid. IR (KBr 1%, *ν*_max⁡_ cm^−1^) 1571 (C=C); 1740–1667 (C=O); ^1^H NMR (CDCl_3, _300 MHz, *δ* ppm): 8.49 (s 1H, NH); 7.37 (s 1H, CH=); 8.35 (s 1H H_(2)_) 7.92 (d 1H H_(4)_, *J* = 8.0); 7.54 (t, 2H H_5_, *J* = 7.72); 7.77 (d 1H H_6_ *J* = 7.7). ^13^C NMR (Acetone-d_6_, 75.4 MHz, *δ* ppm): 169.86 (C=O_2_); 166.88 (C=O_4_); 119.55 (C_5_ heterocycle); 143.65 (CH=); 136.25 (C_1_); 123.06 (C_2_); 148.74 (C_3_); 122.14 (C_4_); 130.16 (C_5_); 131.91 (C_6_). Anal. Calcd. for C_10_H_6_N_2_O_4_S: C, 48.00%; H, 2.42%; N, 11.20%. Found: C, 48.35%; H, 2.1%; N, 11.01%; HRMS^+^: calcd 250.0048; found 250.0051. 

*5-(4-Nitro-arylidene)-thiazolidine-2,4-dione (**2h**)*. Yield 69%; mp 220–223°C; Rf 0.52 (CHCl_3_/MeOH 9.6 : 0.4). Recrystallization: THF; IR (KBr 1%, *ν*_max⁡_ cm^−1^) 1575 (C=C); 1734–1676 (C=O). ^1^H NMR (CDCl_3, _300 MHz, *δ* ppm): 8.49 (s 1H, NH); 7.74 (s 1H, CH=); 7.63 (d 1H, H_(2)_–H_(6)_ *J* = 8.65); 8.09 (d 1H (H_(3)_–H_(5)_ *J* = 8.65). ^13^C NMR (Acetone-d_6_, 75.4 MHz, *δ* ppm): 169.86 (C=O_2_); 166.89 (C=O_4_); 119.50 (C_5_ heterocycle); 142.55 (CH=); 141.50 (C_1_); 129.37 (C_2_)–(C_6_); 124.13 (C_3_)–(C_5_); 147.21 (C_4_); Anal. Calcd. for C_10_H_6_N_2_O_4_S: C, 48.00%; H, 2.42%; N, 11.20%. Found: C, 48.35%; H, 2.1%; N, 11.01%; HRMS^+^: calcd 250.0048; found 250.0049. 

*5-(3-Chloro-arylidene)-thiazolidine-2,4-dione (**2i**)*. Yield 54%; mp 208-209°C; Rf 0.46 (Hexane/Ethyl acetate 4 : 1). Recrystallization: ethyl acetate; IR (KBr 1%, *ν*_max⁡_ cm^−1^) 1582 (C=C); 1729–1672 (C=O). ^1^H NMR (CDCl_3, _300 MHz, *δ* ppm): 8.48 (s 1H, NH); 7.77 (s 1H, C=H); 7.34 (s 1H_(2)_); 7.18 (d 1H_(4)_ (*J* = 8.07); 7.36 (t 2H_(5)_ (*J* = 8.07); 7.36 (d 1H_(6)_ *J* = 2.27); ^13^C NMR (Acetone-d_6_, 75.4 MHz, *δ* ppm): 169.86 (C=O_2_); 166.46 (C=O_4_); 119.55 (C_5_ heterocycle); 143.09 (CH=); 135.81 (C_1_); 127.72 (C_2_); 133.85 (C_3_); 128.62 (C_4_); 130.25 (C_5_); 125.78 (C_6_). Anal. Calcd. for C_10_H_6_ClNO_2_S: C, 50.11%; H, 2.52%; N, 5.84%. Found: C, 50.55%; H, 2.67%; N, 5.57%. HRMS^+^: calcd 238.9808; found 238.9810.

The compounds (**2a**–**i**) were synthesized by Knoevenagel condensation with nine aromatic aldehydes yielding 5-arylidene-thiazolidine-2,4-dione derivatives. The infrared spectrum of these compoundsshowed a strong absorption band of the functional group ranging between 1566 at 1775 cm^−1^ corresponding to C=C and 1775–1676 cm^−1^ concerning the carbonyl in positions 2 and 4 of the thiazolidine-2,4-dione ring [25]. The molecular structures of the compounds were established by ^1^H NMR and ^13^C NMR spectra which exhibited the presence of the signals corresponding to hydrogen and carbons in the molecule. The compound (**2a**) presented a singlet at 3.77 ppm of 3 hydrogen referent to OCH_3 _and a singlet at 6.02 concerning the absorption of the OH group. This substituent also presented absorption at 56.50 ppm of the OCH_3_ in ^13^C NMR. The group OCH_3 _in (**2d**) presented signal in 3.77 ppm to hydrogen and 56.50 to absorption in ^13^C NMR. The absorption of hydrogen as a singlet at 3.65 ppm of the OCH_3_ group at position 4 of arylidene and 55.67 to ^13^C NMR was sufficient to characterize the compound (**2e**). The compound (**2f**) presented absorption as a singlet at 2.36 ppm to hydrogen in methyl group at position 3 and 20.15 ppm to ^13^C NMR in the molecule. The ^1^H NMR spectra of compounds showed only one kind of proton absorption for CH= in the range 7.71–7.78. According to the literature, this proton absorption is the compound represented by the configuration *Z*, as illustrated by the absorption of the CH= group. Isomer (*Z*) appears to be more thermodynamically stable than isomers *E* [26].

The ^1^H NMR spectrum of compounds in CDCl_3_-d6 revealed the presence of methyne moiety and the position of the hydrogen of the aromatic group. In addition, the structures were supported by their mass spectrum which revealed a molecular ion peak. The elemental analysis was also important to supplement the characterization of the chemical structure.

### 3.2. Antimicrobial Activity

The results of the disc diffusion test are shown in Table 1. Most of the synthesized compounds were active against Gram-positive bacteria. MIC and MBC values are shown in Table 2.

Compound (**1**) thiazolidine-2,4-dione showed no antimicrobial activity against any of the tested microorganisms [12]. When it reacted with aromatic benzaldehydes forming derivatives substituted at position 5, however, all derivatives showed antimicrobial activity. This affirmation is similar to what is in the literature, showing that substitution at position 5 with arylidene groups has an important effect on enhancing the antimicrobial properties [14].

This shows the importance of the dual CH= bond at position 5, thus introducing a pharmacophore group in the molecule. This group generally influences the specific structural changes in biological effects, stereochemistry, size, and shape of the carbon chain as well as the nature of the other substituent. The compound (**2a**) with substituents 3-OCH_3_ and 4-OH of the arylidene ring was the most active of all, showing better inhibition for almost all microorganisms tested. This compound inhibited all Gram-positive bacteria tested and, additionally, was the only one that inhibited the growth of Gram-negative bacteria, alcohol acid resistant bacteria, and also the yeast, as shown in Table 1. Generally, 4-thiazolidinediones derivatives have demonstrated antimicrobial activity against Gram-positive and Gram-negative bacteria [14].

Compounds (**2b**) and (**2c**), containing the substituents 2,4-Cl_2_ and 3,4-Cl_2_, respectively, inhibited all Gram-positive bacteria tested and also the alcohol acid resistant bacteria. However, compound (**2c**) showed halos slightly smaller than (**2b**).

The compound (**2d**) with 4-OH group was active only for two Gram-positive bacteria,* M. Luteus* and* E. faecalis*, while (**2e**), substituted with 3-OCH_3_, was only active against* M. luteus*. This fact indicates that the groups OH and OCH_3_ separately do not contribute to a significant antimicrobial activity. This fact is reinforced by the activity of compound (**2a**), which had both groups (3-OCH_3_ and 4-OH), against all tested microorganisms, including the Gram-negative bacterium* P. aeruginosa*. The compounds (**2f**,** 2g**, and** 2h**) were active only for Gram-positive bacteria, except* E. faecalis*,with halos between 17.8 ± 0.62 and 23.8 ± 0.16 mm. The compound (**2i**) also inhibited the growth of all Gram-positive bacteria.

In general, all compounds tested were active against Gram-positive bacteria presenting values of 16 and 32 *μ*g/mL for* S. aureus* and 4 and 8 *μ*g/mL for* M. luteus* and* B. subtilis*, for MIC and MBC, respectively. MIC and MBC values for* M. smegmatis* were 16 and 32 *μ*g/mL, smaller than the standard drugs. The compounds (**2b**,** 2c**,** 2g**,** 2h**, and** 2i**) showed MIC and MBC values for* S. aureus* near those obtained for the standard drug, cefalexin. The compounds (**2b**) and (**2c**) showed MIC and MBC for* M. Luteus *and* B. subtilis *also near the standard drug. The results for* M. smegmatis* 16 and 32 *μ*g/mL were lower than the standard Kanamycin (128 *μ*g/mL) showing significant activity in inhibiting these microorganisms. Recent studies with 5-hydroxy-arylidene derivatives showed MIC values higher than 128 *μ*g/mL for two Gram-negative bacteria,* E. coli* and* P. aeruginosa*, and 128 *μ*g/mL for* E. faecalis* and* S. aureus* [7].

These results showed that these substituents separately did not have significant antimicrobial activity. The groups chlorine, nitro, and methyl at position 3 on the thiazolidine ring seem to favor this activity for other positions [7]. This affirmation reveals that electrons donating or withdrawing groups in molecules increase the lipophilicity of the compounds, which alters the permeability across the bacterial cell membrane [27]. These molecules showed the greatest inhibition among the tested compounds. The compounds (**2b**,** 2c**, and** 2i**) containing chlorine atoms in the molecule were active for all Gram-positive bacteria, indicating the power of chlorine in antimicrobial activity.

This demonstrates that the substitution of arylidene at position 5 on the thiazolidine ring enhances the antimicrobial activity of the pharmacophore group in these compounds. The condensation of the compounds 5-arylidene-thiazolidine-2,4-dione was crucial to generate considerable activity in the synthesized derivatives [8]. The results of this study demonstrate significant antimicrobial activity for compounds 5-arylidene-thiazolidine-2,4-dione substituted (**2a**–**i**), with values similar to the drugs tested cephalexin >128, confirming the antimicrobial activity of thiazolidine compounds as described in the literature [1, 27]. These compounds probably act on the synthesis of peptidoglycan from the cell wall, which explains the higher antimicrobial effects on Gram-positive bacteria instead of Gram-negative ones [2, 28].

## 4. Conclusion

In order to find an antimicrobial agent, nine compounds were synthesized and tested against Gram-positive, Gram-negative, and alcohol acid resistant bacteria and yeast. All compounds were active against all Gram-positive bacteria. One of the compounds was the most active, inhibiting Gram-positive, Gram-negative, and alcohol acid resistant bacteria and also the yeast. The other compounds had varied activity between classes of Gram-positive and acid alcohol resistant bacteria. Compounds containing chlorine in the molecule showed the best antibacterial activity, thus demonstrating the power of this atom on the bacteria. The compounds with substituents nitro and methyl also showed significant activity. All the compounds presented the *Z* configuration. The chemical structures of the compounds were determined by physical methods IR, ^1^HNMR, ^13^CNMR and mass spectrometry.

## Figures and Tables

**Table 1:** Antimicrobial activity of compounds tested against different microorganisms (diameter of inhibition halo in mm).

		Microorganisms								
Synthetized compounds (300 µg/disc)		Gram-positive bacteria				Gram-negative bacteria			Alcohol acid resistant bacteria	Yeast
*n *	Subs.	*S. aureus* DAUFPE 01	*M. luteus* DAUFPE 06	*E. faecalis* DAUFPE 138	*B. subtilis* DAUFPE 16	*P. aeruginosa* DAUFPE 39	*E. coli* DAUFPE 224	*S. marcescens* DAUFPE 398	*M. smegmatis* DAUFPE 71	*C. albicans* DAUFPE 1007
**1**	—	0	0	0	0	0	0	0	0	0
**2a**	3-OCH_3_ 4-OH	19.3 ± 0.47	27.8 ± 0.16	11.2 ± 0.62	15.0 ± 0.16	15.0 ± 0.16	0	0	19.3 ± 0.77	16.2 ± 0.82
**2b**	2,4-Cl	11.7 ± 0.56	32.0 ± 0.81	18.8 ± 0.85	30.7 ± 0.47	0	0	0	19.2 ± 0.41	0
**2c**	3,4-Cl	19.4 ± 0.08	34.0 ± 0.80	8.4 ± 0.17	25.4 ± 0.05	0	0	0	12.1 ± 0.09	0
**2d**	4-OH	0	13.8 ± 0.56	11.0 ± 0.82	0	0	0	0	0	0
**2e**	3-OCH_3_	0	12.7 ± 0.56	0	0	0	0	0	0	0
**2f**	3-CH_3_	18.3 ± 0.47	0	18.8 ± 0.16	17.8 ± 0.62	0	0	0	0	0
**2g**	3-NO_2_	21.8 ± 0.16	0	22.8 ± 0.70	23.7 ± 0.16	0	0	0	0	0
**2h**	4-NO_2_	23.8 ± 0.16	0	21.9 ± 0.17	18.6 ± 0.15		0	0	0	0
**2i**	3-Cl	20.8 ± 0.27	24.8 ± 0.16	21.8 ± 0.16	27.7 ± 0.16	0	0	0	0	0

Kan (30 *μ*g/disc)		23.0 ± 0.82	28.3 ± 0.09	0	14.0 ± 0.82	20.0 ± 0.12	15.1 ± 0.19	15.0 ± 0.12	40.0 ± 0.12	—
Cef (30 *μ*g/disc)		35.3 ± 0.09	54.0 ± 0.12	24.1 ± 0.19	45.0 ± 0.12	0	24.3 ± 0.09	0	0	—
Ket (300 *μ*g/disc)		—	—	—	—	—	—	—	—	24.1 ± 0.19

Standard compounds: Cef: cephalexin; Ket: ketoconazole; Kan: kanamycin.

—: not tested; *X*: medium; *δ*: standard deviation.

**Table 2:** Inhibition and bactericidal concentrations of compounds (**2a–i**), with respect to different microorganisms (*μ*g/mL).

Synthetized compounds	Microorganisms											
	*S. aureus* DAUFPE 01		*M. luteus* DAUFPE 06		*B. subtilis* DAUFPE 16		*M. smegmatis* DAUFPE 71		*E. faecalis* DAUFPE 138		*C. albicans* DAUFPE 1007	
	MIC	MBC	MIC	MBC	MIC	MBC	MIC	MBC	MIC	MBC	MIC	MBC
**2a**	16	32	4	8	4	8	16	32	64	>128	8	32
**2b**	8	16	2	4	2	4	2	4	16	32	—	—
**2c**	8	16	2	4	2	4	2	4	16	32	—	—
**2d**	—	—	4	8	—	—	—	—	—	—	—	—
**2e**	—	—	4	8	—	—	—	—	—	—	—	—
**2f**	16	32	16	32	4	8	—	—	—	—	—	—
**2g**	8	16	8	16	16	32	—	—	—	—	—	
**2h**	8	16	8	16	—	—	—	—	—	—	—	—
**2i**	8	16	16	32	4	8	—	—	—	—	—	—

Cefalexin	8	16	<2	2	<2	<2	>128	>128	>128	>128	—	—
Ketoconazole	—	—	—	—	—	—	—	—	—	—	<2	2

MIC: minimum inhibitory concentration;

MBC: minimum bactericidal concentration;

—: not tested.
